# Enhancing the Performance of a Metal-Free Self-Supported Carbon Felt-Based Supercapacitor with Facile Two-Step Electrochemical Activation

**DOI:** 10.3390/nano12030427

**Published:** 2022-01-27

**Authors:** AlBatool A. Abaalkhail, Basheer A. Alshammari, Ghzzai N. Almutairi, Feraih S. Alenazey, Mohammed F. Alotibi, Asma M. Alenad, Abdullah G. Alharbi, Thamer S. Almoneef, Bandar M. AlOtaibi

**Affiliations:** 1 The Center of Excellence for Advanced Materials and Manufacturing, King Abdulaziz City for Science and Technology, Riyadh P.O. Box 6086, Saudi Arabia; aabaalkhail@kacst.edu.sa; 2 Material Science Research Institute, King Abdulaziz City for Science and Technology, Riyadh P.O. Box 6086, Saudi Arabia; bshammari@kacst.edu.sa (B.A.A.); mfalotaibi@kacst.edu.sa (M.F.A.); 3 The National Center for Energy Storage Technologies, King Abdulaziz City for Science and Technology, Riyadh P.O. Box 6086, Saudi Arabia; gmotari@kacst.edu.sa (G.N.A.); Alenazey@kacst.edu.sa (F.S.A.); 4 Chemistry Department, College of Science, Jouf University, Sakaka P.O. Box 2014, Saudi Arabia; amenad@ju.edu.sa (A.M.A.); agalharbi@ju.edu.sa (A.G.A.); 5 Electrical Engineer Department, Prince Sattam Bin Abdulaziz University, Alkharj P.O. Box 173, Saudi Arabia; T.almoneef@psau.edu.sa

**Keywords:** carbon felt, supercapacitor, electrochemical activation, carbon electrode, flexible electrode, energy storage

## Abstract

Carbon felt (CF) is an inexpensive carbon-based material that is highly conductive and features extraordinary inherent surface area. Using such a metal-free, low-cost material for energy storage applications can benefit their practical implementation; however, only limited success has been achieved using metal-free CF for supercapacitor electrodes. This work thoroughly studies a cost-effective and simple method for activating metal-free self-supported carbon felt. As-received CF samples were first chemically modified with an acidic mixture, then put through a time optimization two-step electrochemical treatment in inorganic salts. The initial oxidative exfoliation process enhances the fiber’s surface area and ultimately introduced oxygen functional groups to the surface, whereas the subsequent reduction process substantially improved the conductivity. We achieved a 205-fold enhancement of capacitance over the as-received CF, with a maximum specific capacitance of 205 Fg^−1^, while using a charging current density of 23 mAg^−1^. Additionally, we obtained a remarkable capacitance retention of 78% upon increasing the charging current from 0.4 to 1 Ag^−1^. Finally, the cyclic stability reached 87% capacitance retention after 2500 cycles. These results demonstrate the potential utility of electrochemically activated CF electrodes in supercapacitor devices.

## 1. Introduction

The electrical double-layer capacitors are electrical energy storage devices that offer attractive features, such as fast storing/releasing of energy, long cycling life, and high reversibility as they combine the benefits of both traditional capacitors and rechargeable batteries. They are commonly called supercapacitors [1,2]. Supercapacitors have emerged as preferable energy storage devices due to the specific properties, namely high power density, faster charge–discharge rates, and stable life cycle [3]. A crucial component of supercapacitor devices is the electrode material, which determines key performance parameters such as power, energy density, and stability [4,5,6]. Carbonaceous materials are widely utilized for electrochemical applications, particularly concerning environmental and energy purposes, and hence, are used as electrodes for supercapacitor applications. In particular, carbon onions, carbon nanotubes (CNTs), graphene, and carbon porous materials are favorable as electrodes as they have a large surface area, high conductivity, and chemical stability [3,5,6,7,8,9,10,11,12,13,14,15].

However, many carbon-based supercapacitors have poor rate capabilities. This limitation is attributable to a range of factors, including the high resistance of the electrode material, its limited ion–diffusion rate, and the substantial interface resistance between conducting substrates and active electrochemical materials [16,17,18,19]. Furthermore, many carbon materials (such as graphene and CNTs) feature relatively high production costs due to the complexity of their fabrication processes; this prevents the large-scale commercialization of such materials in practical applications [20]. Moreover, despite the considerable body of work on facilitating graphene, there are still challenges in controlling its quality and quantity, which impedes its commercialization in supercapacitor applications [21].

The high commercial suitability of carbon felt (CF) has attracted research interest for further improving their utility by increasing surface area and improving surface mechanisms for supercapacitor applications. Most of the reported studies use carbon felt as a supporting substrate for other active supercapacitive materials, such as metal oxides. Few studies have explored activation methods to enhance the performance of stand-alone carbon felt as a supercapacitor electrode. Notably, CF is a fibrous material that features a low production cost. CFs have been widely used as electrodes in diverse applications, such as redox-flow batteries and electrochemical oxidation for wastewater treatment; they feature excellent conductivity and numerous redox reaction sites as a consequence of their high surface-to-volume ratio [22]. However, CFs are also characterized by hydrophobic surfaces, which limit the penetration of electrolyte ions, and so limit the material’s charge storage capabilities [22]. That is, untreated CF exhibits a low specific capacitance of 3 Fg^−1^, which is slightly higher than carbon cloth (1–2 Fg^−1^), but considerably inferior to both graphene (100–200 Fg^−1^) and single-walled carbon nanotubes (100–200 Fg^−1^) [20,23]. Thus, activation or functionalization of CF is required to utilize its dense surface. A specific capacitance of 280 Fg^−1^ at 0.5 mA cm^−2^ has been obtained by treating CF with a freeze-drying assisted KOH activation method [20]. Still, despite the success of this method, there are issues regarding the cost effectiveness of its activation processes. Specifically, the reported process requires extreme temperatures and pressure, in addition to a long processing time; these requirements restrict its scalability and cost effectiveness. Most of these enhancement techniques add some complexity and present issues for scaling up electrode fabrication, thereby increasing electrode cost and negating the benefits of carbon felt as an inexpensive material. Hence, the development of low-cost, self-supporting electrode materials is crucial for mitigating the technical issues and real market barriers that hinder practical use of supercapacitor devices. 

It is worth mentioning that CF capacitance can be readily improved through functionalizing its surface by means of incorporating heteroatoms such as nitrogen and oxygen, in which case the extra capacitance is supported by redox reactions. That is, surface functional groups, such as those containing oxygen, phosphorus, and nitrogen can essentially increase capacitance by providing pseudocapacitance effects and enhancing the wettability of porous carbon when in electrolytes [24,25,26,27,28]. For instance, the higher capacitance in graphene oxide (GO) relative to graphene is attributed to pseudocapacitance resulting from oxygen-containing functional groups [29]. Similarly, functionalizing a carbon fiber material, such as carbon cloth, with oxygen functional groups can enhance its electrode capabilities [30]. Additional findings suggest that the reduction of these functional groups provides further improvement to capacitance [23,31]. Therefore, functional groups have a distinct and noticeable impact on the capacitive behavior of graphene- and carbon-based electrodes in supercapacitor devices.

In this study, we attempted to address the costly processing of CF (a low-cost raw material) in order to achieve cost effectiveness. We investigated an activation route by which to exfoliate and ultimately introduce oxygen functional groups, along with consequent effects on the CF material’s performance as an electrode for supercapacitor devices. Briefly, as-received CFs were cleaned, immersed in an acidic mixture, and subsequently treated with an optimized two-step electrochemical method, i.e., oxidation, followed by a reduction in inorganic salts. A significant 205-fold improvement in performance was achieved, with a specific capacitance of 205 Fg^−1^ obtained at a 23 mAg^−1^ current density in a three-electrode configuration. Considering the relatively low cost of CF and the significant capacitive improvement realized by our treatment, this material has potential for use in commercial high-performing supercapacitor devices. This work clearly illustrates the extraordinary potential of self-supported carbon-based electrodes for use in supercapacitor devices. Through this fast and efficient activation treatment (~2 h treatment time with very low energy consumption), real-time applications and commercialization of supercapacitors can be realized.

## 2. Materials and Methods

### 2.1. Sample Preparation

All chemicals for CF treatment were analytical grade and purchased from Sigma Aldrich (Gillingham, UK). Before the treatment process, the as-received CF (purchased from SGL Carbon, Wiesbaden, Germany) was cut into small pieces (1 × 1 cm^2^, mass per unit area 17 mg/cm^2^) and were cleaned with a mixture of acetone and isopropyl alcohol 1:1 (*v*/*v*), followed by thorough washing with deionized water. Afterward, samples were soaked for a few hours in a combination of 3:1 (*v*/*v*) sulfuric acid (H_2_SO_4_) and nitric acid (HNO_3_), then rinsed generously with deionized water until an absence of sulfate was achieved.

**Figure 1:**
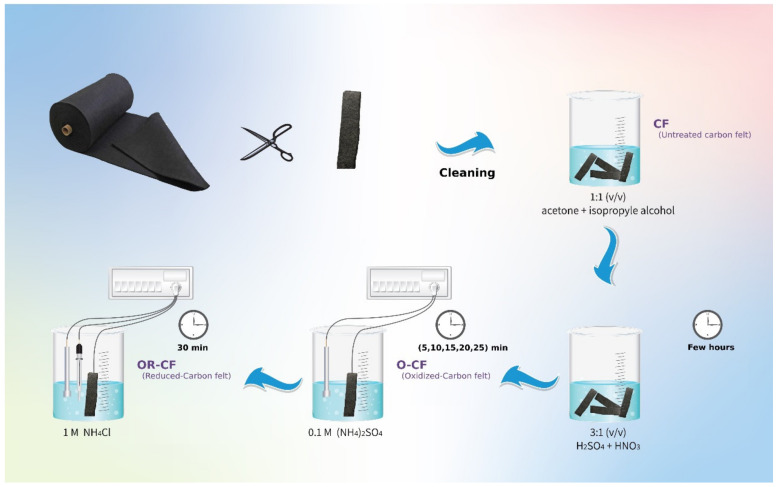
Schematic diagram of the activation of carbon felt using wet chemical and electrochemical methods with H_2_SO_4_/HNO_3_ and (NH_4_)_2_SO_4_/NH_4_Cl, respectively.

As illustrated in Figure 1, the electrochemical oxidative process was carried out using a two-electrode system in 0.1 mol/L aqueous solution of ammonium sulfate ((NH_4_)_2_SO_4_) with a platinum mesh as the cathode and a CF electrode as the anode; different oxidizing times were optimized (5, 10, 15, 20, and 25 min) at a potential of 10 V vs. cathode. After oxidation, the CF was reduced in 1 mol/L of ammonium chloride (NH_4_Cl) solution for a fixed time of 30 min using a 3-electrode configuration with a saturated calomel electrode (SCE) as the reference electrode, platinum mesh as the counter electrode, and treated carbon felt as the working electrode, operated at a potential of −1.24 V vs. SCE. The treated electrodes were rinsed thoroughly with deionized water and dried at room temperature. In this study, 15 min was found to be the optimum oxidizing time for preparing carbon felt electrodes; thus, that period was used for producing all samples subjected to characterization techniques, which included *scanning electron microscopy* (SEM), transmission electron microscopy (TEM), the Brunauer–Emmett–Teller (BET) method for determining surface area, Fourier transform infrared spectroscopy (FTIR), Raman spectroscopy, and X-ray photoelectron spectroscopy (XPS). In the following sections, CF denotes the untreated carbon felt samples, O-CF refers to the carbon felt oxidized for 15 min, and OR-CF represents the sample that underwent 15 min of the oxidative process followed by the reduction process. Other samples underwent different oxidative durations and are denoted as OR-X, with X being the oxidation time (5, 10, 15, 20, or 25 min).

### 2.2. Sample Characterization

The morphology of OR-CF and CF samples was characterized using scanning electron microscopy (SEM, Jeol JSM-6010PLUS/LV, Peabody, St. Louis, MO, USA) and transmission electron microscopy (TEM, FEI Talos F200, Thermo Scientific, Waltham, MA, USA). Surface area was characterized using the Brunauer–Emmett–Teller method (BET, ASAP 2420 V2.09, micromeritics, Norcross, GA, USA). Additionally, the presence of surface functional groups on OR-CF, O-CF, and CF was characterized by Fourier transform infrared spectroscopy (FTIR, Thermo-Fisher FTIR Nicolet iS10 and a DTGS detector Waltham, MA, USA), Raman spectroscopy (Raman, Thermo-Fisher DRX2 Raman spectrometer, Thermo Scientific, Waltham, MA, USA), and X-ray photoelectron spectroscopy (XPS, Kratos Axis SUPRA, Kratos Analytical Ltd, Manchester, UK). Finally, electrochemical evaluations were carried out with an Autolab PGSTAT302N (Metrohm, Sharjah, United Arab Emirates) at room temperature in a 3-electrode scheme using 1 M H_2_SO_4_ as the electrolyte, platinum as the counter electrode, and SCE as the reference electrode.

### 2.3. Calculations

The specific gravimetric capacitance of the treated electrodes was calculated from cyclic voltammograms as

(1) $$C_{s}=\frac{\int_{0}^{2V_{0}/v}\left|i\right|\;dt}{2V_{0\;}M}.$$

where $C_{s}$ is the gravimetric specific capacitance in Fg^−1^, $V_{0}$ is the potential window in volts, v is the scan rate in Vs^−1^, i is the current in amps, and *M* is the mass of the electrode in grams. A second determination of specific capacitance was calculated from the galvanostatic charge–discharge curves with inclusion of IR drop using

(2) $$C_{s}=\frac{I_{dis}\;\Delta t_{V_{0}-2V_{0}}}{V_{0}\;M}$$

and without the IR drop by adjusting the same equation to give [32]

(3) $$C_{s}=\frac{I_{dis}\;\Delta t_{V_{0}-2V_{0}}}{\left(V_{0}-V_{IR}\right)\;M}$$

where $V_{IR}$ is the voltage drop on the electrode.

## 3. Results and Discussion

**Figure 2:**
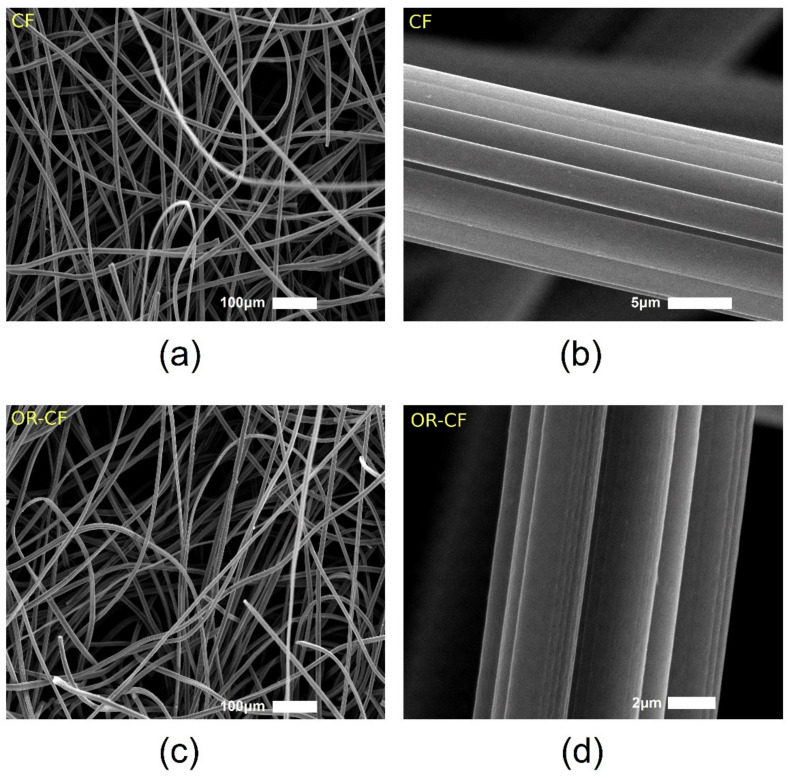
SEM micrographs of (**a**) CF fibers, (**b**) the CF surface at a five-micrometer scale, (**c**) OR-CF fibers, and (**d**) the OR-CF surface at a two-micrometer scale.

The nature of the (CF) surface and exfoliation of the fibers upon oxidative treatment were investigated using SEM images, specifically comparing the morphology of as-received CF and treated carbon felt (OR-CF). As evidenced in Figure 2a,b, as-received CF possessed an entangled fiber network with a smooth surface. In contrast, as shown in Figure 2c,d, the 3D structure of the OR-CF showed less entanglement and a rougher surface with cracks resulting from the oxidative treatment. For further morphological investigation, high-resolution TEM images were taken of the edges of single-fiber filaments.

**Figure 3:**
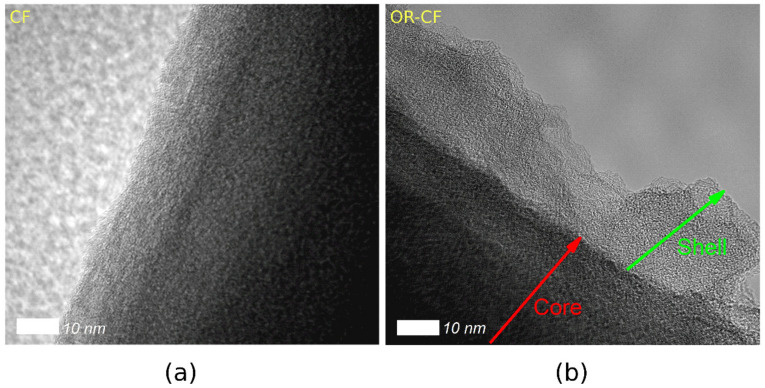
High-resolution TEM micrographs illustrating the exfoliation of carbon felt at fiber edges: (**a**) CF at a ten-nanometer scale and (**b**) the core–shell structure of OR-CF.

Figure 3a demonstrates that fibers of the as-received CF are more intact and exhibit smoother surfaces, whereas Figure 3b clearly illustrates the exfoliation of the OR-CF fibers that results from electrochemical treatment. This exfoliation produced a core shell-like structure with an approximate shell thickness of 30 nm. More figures showcasing the morphological differences of these samples are provided in the supporting information (Figures S1 and S2). Ultimately, these microscopic observations indicate that the OR-CF surface was substantially altered by two-step electrochemical treatment. Previous reports have evidenced similar morphologies for activated carbon cloth, carbon fibers, and carbon felt [23,33,34,35,36]; thus, electrochemical exfoliation of carbon materials generally leads to a significant improvement in electrode properties, including surface area.

To elucidate the properties of CF subjected to the aforementioned treatment, surface area analysis was carried out using the BET method. The OR-CF sample exhibited a surface area of ~45 m^2^ g^−1^, which was 68 times higher than that of the as-received CF (~0.66 m^2^ g^−1^). The magnitude of this increase is attributed to the exfoliation that occurred during both chemical oxidation and the electrochemical oxidative processes; that is, the intense chemical/electrochemical treatments created mesopores, micropores, and flakes on the surfaces of the material. These outcomes are in good agreement with the morphological interpretations shown in Figure S3.

Notably, the surface area increase observed for OR-CF is almost double that achieved for activated carbon cloth using a different activation process [36], despite both studies using similar chemical treatments and conditions. Many other studies have reported moderate improvements in the BET surface area of activated carbon fiber, cloth, or even felt [20,23,36,37,38]. For instance, one study that chemically modified carbon cloth by applying a mixture of concentrated H_2_SO_4_, HNO_3_, and KMnO_4_ followed by a reduction process obtained a surface area 11.5 times the initial value [23]. Differences in BET surface area values across those studies could be attributed to differences in the modification method applied, related conditions, and type of used raw materials. 

Although surface area is the main factor in determining double-layer capacitance in carbon-based electrodes, other factors, such as electrical conductivity, surface functionalities, structure, pore size, and particle size also affect the capacitance and final performance of supercapacitors. In this study, OR-CF was found to mainly consist of mesopores with an average pore width of 2 nm, which is less than the average pore size for carbon cloth (~200 nm) reported by other studies [36]. This is mainly attributable to the dense fiber network characteristic of CF materials. Importantly, pore size is crucial for ion diffusion inside carbon materials, and the pore size observed with treated CF still permits access to hydrated ions [29]. Ultimately, carbon materials having both good surface area and good electrolyte accessibility permit the transport of charge and optimal storage [22,39,40].

**Figure 4:**
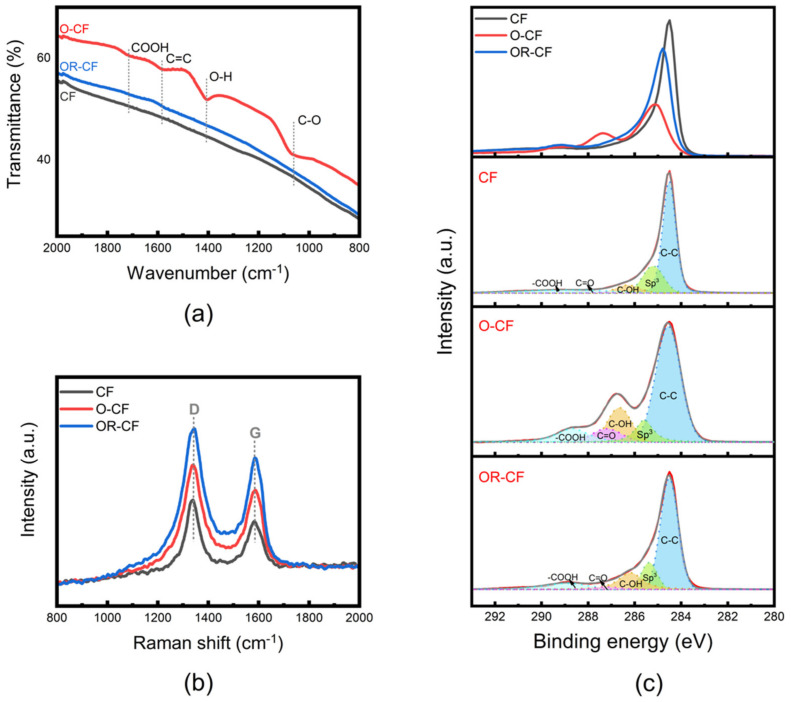
Characterization of CF, O-CF, and OR-CF in terms of (**a**) FTIR spectra and the corresponding oxygen functional groups, (**b**) Raman spectra, and (**c**) high-resolution XPS, specifically the normalized C1s spectra of CF, O-CF, and OR-CF. Red curves are the experimental results that were deconvoluted into five synthetic peaks (dashed lines). The black curve is the summation of those synthetic peaks.

Fourier-transform infrared spectroscopy (FTIR) was used to confirm the presence of functional groups upon modification of CF, the results of which are shown in Figure 4a. It is clear that in contrast to the nearly featureless spectrum of CF, the O-CF sample shows several peaks in the range of 1057 to 1715 cm^−1^. These peaks are usually characteristic of primary alcohol, being ether (–C–O), carboxyl (–COOH), carbonyl (–C=O), or hydroxyl (–OH) functional groups. The FTIR peaks observed in this study and their corresponding functional groups are specified in Table S1 in the Supplementary Materials. Of particular interest is the peak located between 1710–1730 cm^−1^, which according to the literature is due to the stretching vibrations of carboxyl groups [41,42]; these groups are active in numerous chemical reactions, thus of great functional importance. In addition, it is likely that HNO_3_ introduces nitrogen functional groups to the O-CF surface, as a peak for N–H bending is observed; however, after the electrochemical reduction to obtain OR-CF, the FTIR spectrum nearly returned to that of untreated CF. All told, the FTIR findings indicate effective modification of the CF surface by HNO_3_, H_2_SO_4_, and (NH_4_)_2_SO_4_.

It is worth noting that FTIR analysis of carbon fibers is reportedly complicated by severe scattering [33]. Therefore, to ensure a thorough investigation into the presence of functional groups and to confirm the efficacy of the modification (or activation) processes, Raman and X-ray photoelectron spectroscopy (XPS) were also used to characterize the surface properties of OR-CF, O-CF, and CF samples. Figure 4b presents the Raman spectra collected for each material. Both O-CF and OR-CF exhibit broader G- and D-band peaks, along with an upshift relative to CF. The calculated I_D_/I_G_ ratio of O-CF and OR-CF increased from 2.02 to 2.17, indicating that the electrochemical treatment results in the enhancement of an OR-CF surface disorder degree and reduces the in-plane sp^2^ domains [23,36]. 

As expected, XPS verified that the treated CF was functionalized with chemical groups containing mainly carbon and oxygen elements. Specifically, the oxygen to carbon ratio (O/C) increased from 4.9% (in CF) to 39.6% (in O-CF) upon the acid and electrochemical oxidative treatments. After the reduction process, the oxygen content reduced to 14.9% in OR-CF, indicating some residual oxygen functional groups on the surface despite the restoration of electrical conductivity. More detailed information about the types of functional groups present was obtained from high-resolution XPS analysis, shown in Figure 4c. The O-CF sample exhibited a broader peak than CF and OR-CF, suggesting that some areas on its surface featured chemical states involving different types of bonding for C1s atoms; these contributed to oxygen formation at the surface. In addition, the blue curve describing OR-CF suggests that the oxygen functional groups created in the oxidative process were all eliminated after the reduction process. Moreover, deconvolution of the C1s spectra for CF, O-CF, and OR-CF revealed several peaks linked to carbon structure and oxygen–carbon binding. In all samples, the C1s signals exhibited prominent peaks at 284.0–285.0 eV; these are typically ascribed to carbon (sp^2^ or sp^3^). This prominent peak is somewhat due to the intrinsic asymmetry of the graphite peak. Other notable peaks around binding energies of 284.0–285.0, 286.0–287.0, 287.5–288.1, and 288.7–288.9 eV are respectively associated with groups containing (–C–C or C=C), (–C–OH), (–C=O), and (–COOH); these types of functional groups have typically been observed in previous reports [23,35,36,38,43,44]. In addition, high-resolution O1s spectra, shown in Figure S5, were obtained to ascertain the oxygen species present after treatment. Two peaks ranging between 532.4–535.6 eV were evident; such peaks are usually related to (–C=O) and (–C–OH) groups.

Overall, the obtained XPS results are in good agreement with the results obtained from the FTIR and Raman techniques, as presented above. All three spectroscopy techniques revealed the presence of some categories of oxygen functional groups as well as carbon bases, through which surface chemistry can be readily connected to the pseudocapacitive behavior of activated carbon materials. However, these functional groups are also responsible for the deterioration of electrical conductivity after oxidation. When the carbon materials are chemically reduced in the second step, the oxygen functional group content is significantly decreased, which results in some restoration of conductivity [40,45].

**Figure 5:**
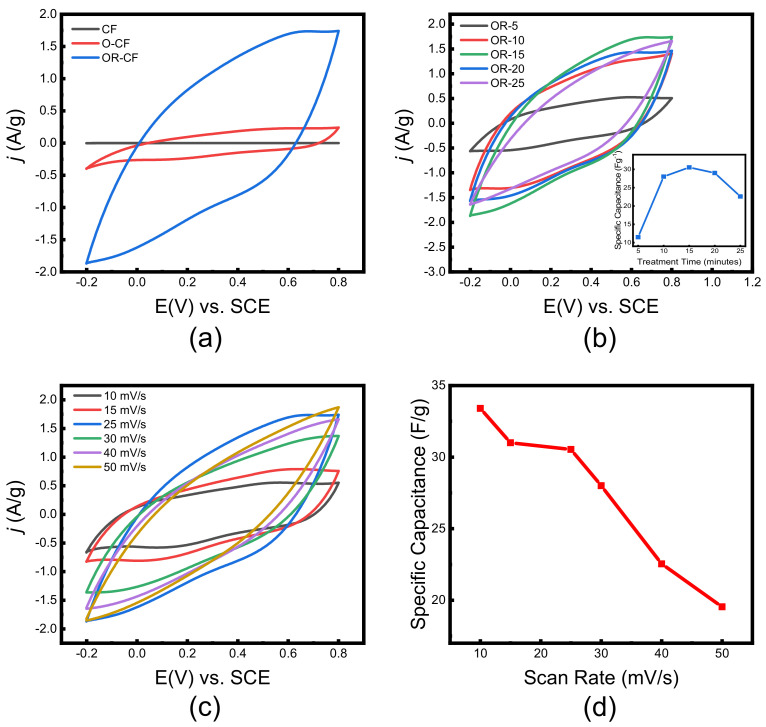
Cyclic voltammetry collected at a scan rate of 25 mV/s in 1.0 M H_2_SO_4_ for (**a**) CF, O-CF, and OR-CF at 15 min oxidizing time, (**b**) OR-CF produced with different oxidizing times (5, 10, 15, 20, and 25 min), (**c**) cyclic voltammetry collected at different scan rates and (**d**) the calculated gravimetric capacitance.

The electrochemical performance of both as-received and treated CF samples was evaluated in a standard three-electrode cell in 1.0 mol/L H_2_SO_4_. Cyclic voltammetry (CV) at a scan rate of 25 mV/s was carried out to investigate the improvement upon each treatment step. The as-received CF possessed inferior capacitive capabilities, evidenced by the nearly overlapping curve in Figure 5a. In contrast, the oxidized sample (O-CF) exhibited clear improvement in terms of increased loop area, attributable to hydrophilicity enhancement, surface area increase, and the addition of functional groups at the surface. However, this enhancement is unsatisfactory as the curve also exhibited a narrow loop due to the resistivity of the O-CF electrode. Upon electrochemical reduction, the OR-CF curve exhibited higher current density, attributable to the enhanced conductivity of the sample, along with a symmetric quasi-rectangular shape, suggesting pseudocapacitive contribution to the electrode’s performance. The pseudocapacitive behavior of potentials between 0.15 and 0.6 V can be ascribed to the transformation of C=O to C–OH, which further proves the presence of residual oxygen functional groups even after the reduction treatment [28].

Ultimately, the OR-CF material achieved a six-fold improvement in capacitance (from 5 to 33.5 Fg^−1^) relative to O-CF. CV scans were also taken of samples produced under different durations of oxidization (5, 10, 15, 20, and 25 min) and a fixed reduction time of 30 min, depicted in Figure 5b. The inset shows the corresponding capacitance values measured at a scan rate of 25 mV/s, which were used to identify the optimum treatment time. OR-15 possessed the largest loop and maximum current density, thus attaining the best capacitance value; therefore, 15 min was taken as the optimum oxidizing time. A CV scan of OR-15 under various scan rates was evaluated, illustrated in Figure 5c. The curves possessed slightly distorted loops due to factors, such as the slow pseudocapacitive contribution of the oxygen functional groups. Figure 5d depicts the inversely linear relation of specific capacitance with scan rate increment. It should be noted that a maximal specific capacitance of 33.5 Fg^−1^ was attained at a scan rate of 10 mV/s, indicating superior performance over the as-received CF.

**Figure 6:**
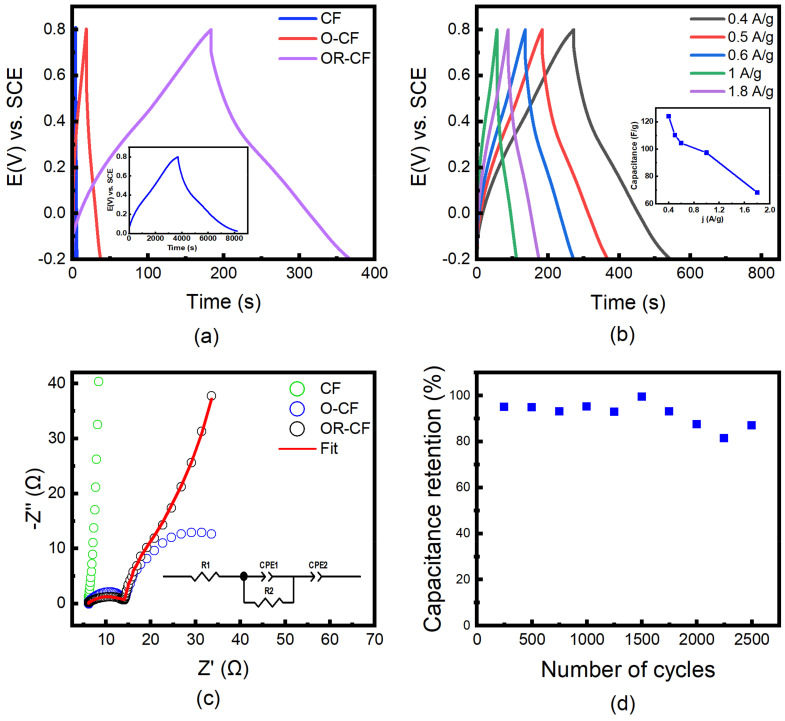
Galvanostatic charge–discharge measurements of (**a**) CF, O-CF, and OR-CF at 0.5 Ag^−1^, the inset depicts the charging/discharging curve at a charging current density of 23 mAg^−1^, resulting in 205 Fg^−1^ and (**b**) OR-CF at different current densities, ranging from 0.4 Ag^−1^ to 1.8 Ag^−1^. The inset represents the specific capacitance relation as current density increases. (**c**) Nyquist plot of CF, O-CF, and OR-CF, along with the fitted circuit measured across a frequency range of 100 kHz to 0.01 Hz. (**d**) Cycling performance of OR-CF at 0.5 Ag^−1^ for 2500 cycles.

In addition, galvanostatic charge and discharge measurements were performed on CF, O-CF, and OR-CF samples at a current density of 0.5 Ag^−1^, the results of which are shown in Figure 6a. All charge–discharge curves exhibited a nearly symmetrical and quasi-triangle shape, indicating a hybrid charge storage performance. The Coulombic efficiency of OR-CF in Figure 6a is 99.5%. The as-received CF clearly demonstrates poor capacitive performance with its extremely short discharge duration of 0.2 s. As further proof of the influence of the efficacy of a reduction step on electrode improvement, OR-CF exhibited a significant improvement in charging/discharging time, 2200 times more than CF and six times more than O-CF; this is attributable to the removal of both unfavorable oxygen functional groups and some surface residues. Ultimately, the difference in performance between O-CF and OR-CF further demonstrates the need for the additional reduction step to improve electrode conductivity.

A study of the relationship between electrode performance and current density was also carried out, obtaining the specific capacitance current density relationship graph shown in Figure 6b. Generally, discharging duration increased as the capacitance value increased or as the charging current decreased. At a charging/discharging current density of 0.4 Ag^−1^, the gravimetric capacitance reached 125 Fg^−1^; when the current density was increased to 1 Ag^−1^, 78% of that value was retained. This indicates a high degree of reliability for the OR-CF electrode at higher charging currents. The treatment enabled the maximal gravimetric capacitance to reach 205 Fg^−1^ at a charging current density of 23 mAg^−1^ shown in the inset of Figure 6a, indicating significant charging capabilities of the treated OR-CF electrode. In comparison with other reports as shown in Table 1, such a simple method clearly illustrates the extraordinary potential of self-supported carbon-based electrodes for use in supercapacitor devices. Through this fast and efficient activation treatment (~2-h treatment time with very low energy consumption), real-time applications and commercialization of supercapacitors can be realized. Table 1 also shows the comparison with carbon cloth as carbon felt and cloth are both made of carbon fibers. However, carbon cloth is comprised of carbon fibers that are woven in an orderly fashion, whereas carbon felt is comprised of unwoven, randomly oriented carbon fibers that result in apparent different textures, as shown below.

To investigate the capacitive mechanisms at work in CF, O-CF, and OR-CF samples, electrochemical impedance spectroscopy was performed, yielding the Nyquist plots illustrated in Figure 6c. The as-received CF had the steepest vertical curve, suggesting a pure capacitive nature that would be advantageous when using CF as an electrode for supercapacitor devices. However, the electrical double-layer capacitance exhibits poor performance as a result of the pores being inaccessible to the electrolyte’s ions, which is attributable to the hydrophobic nature of the as-received CF. Meanwhile, at high frequencies, the charge transfer kinetics and ion dynamics become more prominent due to their quick kinetic nature. From the high frequency in Figure 6c, it is evident that both O-CF and OR-CF experience low electrode series resistance and charge transfer resistance. On the other hand, the midfrequency range can provide information about kinetics associated with pore structure. In contrast with the enhanced kinetics of OR-CF, the additional, incomplete semicircle evident for O-CF indicates poor diffusion of SO_4_^2−^ ions inside the pores accompanied by larger charge resistance, a characteristic of unfavorable oxygen functional groups and the residual hydrophobic surface of the CF [53]. The reduction step seems to eliminate this nonideality by improving the hydrophilicity of the sample and removing the residual oxygen functional groups that hindered electrode conductivity. Moreover, at the lower frequency range, ions can fully access the narrow pores, providing more sites for formation of the double layer and allowing the occurrence of dependent kinetics such as faradic reactions. Thus, the vertical line associated with ideal double-layer capacitance becomes more slanted, and the relaxation frequency is shifted toward phase angles lower than 45°, resulting in a circuit fit best described with a constant phase element (CPE), as presented in the inset of Figure 6c [52,54]. The fitted curve for OR-CF yielded the circuit on the graph, values for which are listed in Table S2 in the Supplementary Materials. R1 is the equivalent series resistance, CPE1 and CPE2 are the constant phase elements related to the mixed mechanisms resulting from the presence of both pores and oxygen functional groups, and R2 is the charge-transfer resistance. Finally, a cyclic charge/discharge test was carried out for 2500 cycles at a current density of 0.5 Ag^−1^, in which OR-CF achieved a remarkable capacitance retention of 87%, shown in Figure 6d. This clearly indicates the high reliability of OR-CF electrodes.

## 4. Conclusions

In summary, we present a simple and cost-effective method for the activation of carbon felt as an alternative, low-cost, and ultimately active material for use in supercapacitor devices. An initial acidic treatment improved CF hydrophobicity; the subsequent electrochemical oxidative process exfoliated the surfaces of CF fibers and introduced oxygen functional groups, leading to both increased surface area and deterioration in conductivity. The addition of a further reduction step resulted in significant improvement of the electrode’s performance. In contrast to the existing treatment of CF in literature, where the activation time can reach 48 h and requires very high/low temperatures (−86 and 1200 °C), OR-CF activation time can be reduced to 1 h and at room temperature. Thus, avoiding the complexities and issues, these enhancement techniques are present in scaling up electrode fabrications, which increases electrode cost and negates the benefits of carbon felt as an inexpensive material. Ultimately, a remarkable maximum capacitance of 205 Fg^−1^ at 23 mAg^−1^ was achieved, and the electrode preserved 87% of its capacitance after 2500 cycles at 0.5 Ag^−1^, without the loading of any electrochemically-active materials. These results illustrate the importance of adding a reduction step when activating carbon and suggest great potential for activated carbon felt materials, in particular as high-performing electrodes for supercapacitor devices. In the future, we emphasize that further improvement of the CF surface area would lead to the full utilization of CF as a bare, self-supported electrode for supercapacitor applications.

## Figures and Tables

**Table 1:** Comparison of previous studies’ data with the present study.

Materials	Activation Method	Electrolyte	Cell Configuration	Capacitance Fg^−1^ (or as Stated)	Charging Current Density mAg^−1^ (or as Stated)	Surface Area (m^2^ g^−1^)	Ref.
**Carbon Felt**	Chemical	1 M H_2_SO_4_	3-electrode	205	23	---	Present study
	Freeze drying	1 M H_2_SO_4_	3-electrode	280	0.5 mA cm^−2^	2109	[20]
	Thermal	2 M H_2_SO_4_	3-electrode	213	2 Ag^−1^	177	[46]
	Thermal	2 M KOH	2-electrode	120	CV	1196	[47]
	Thermal	2 M KOH	3-electrode	163	20	1140	[48]
	Thermal	VOSO_4_ and H_2_SO_4_	Cell	234 mFcm^−2^	2 mA cm^−2^	---	[49]
**Carbon Cloth**	Chemical	1 M H_2_SO_4_	3-electrode	8.8 mFg^−1^	20 mA cm^−2^	61.2	[23]
		PVA/H_2_SO_4_	2-electrode	1.55 mFg^−1^			
	Polymerization	1 M H_2_SO_4_	3-electrode	42	6 mA cm^−2^	---	[36]
	Thermal	6 M KOH	2-electrode	79	1 mA cm^−2^	751	[50]
	Thermal	3 M NaBF_4_	2-electrode	3291 mFcm^−2^	power density of 9000 μW cm^−2^	698	[51]
	Plasma	2 M KOH	3-electrode	741 mFcm^−2^	0.5 mA/cm^2^	---	[52]

## Data Availability

Not applicable.

## Supplementary Materials

The following supporting information can be downloaded at: https://www.mdpi.com/article/10.3390/nano12030427/s1. Figure S1. SEM image of OR-CF rough surface; Figure S2. Low-magnification TEM at the edge of carbon felt fiber of untreated carbon felt CF (left) and treated carbon felt OR-CF (right); Figure S3. Nitrogen adsorption isotherm of CF and OR-CF at 77 K; Table S1. FTIR corresponding functional groups contained corresponding to their peaks; Figure S4. FTIR survey of CF, O-CF and OR-CF; Figure S5. XPS O1s spectrum of CF, O-CF and OR-CF; Figure S6. Cyclic voltammetry measurement of electrochemically oxidized carbon felt electrodes O-X (X: oxidizing time (5,10,15,20,25)); Figure S7. Extended electrochemical oxidizing conditions while constant reduction time of 30 minutes. (a) SEM image of OR-120 surface and (b) cyclic voltammetry of rest of the electrodes OR-X with X being corresponding treatment time in seconds; Figure S8. Galvanostatic charge/discharge measurements of OR-CF at 0.023 A g^−1^; Table S2. The corresponding circuit element values of impedance spectroscopy.
