# Supplementary material for: Enhancing the Performance of a Metal-Free Self-Supported Carbon Felt-Based Supercapacitor with Facile Two-Step Electrochemical Activation
Source: Nanomaterials (Basel). 2022 Jan 27;12(3):427. doi: 10.3390/nano12030427 (PMC8838256; doi:10.3390/nano12030427)
Supplement: Supplementary file 1 [file nanomaterials-12-00427-s001.zip › nanomaterials-1539040 supplementary xml pdf corrected/nanomaterials-1539040 supplementary xml pdf corrected.pdf]

nanomaterials

Supporting Information

Enhancing the Performance of a Metal-Free Self-Supported Carbon Felt-Based Supercapacitor with Facile Two-Step Electrochemical Activation

AlBatool A. Abaalkhail [1], Basheer A. Alshammari [2], Ghzzai N. Almutairi [3], Feraih S. Alenazey [3], Mohammed F. Alotibi [2], Asma M. Alenad [4], Abdullah G. Alharbi [4], Thamer S. Almoneef [5] and Bandar M. AlOtaibi [1,3,*]

1 The Center of Excellence for Advanced Materials and Manufacturing, King Abdulaziz City for Science and Technology, P.O. Box: 6086, Riyadh, Saudi Arabia; aabaalkhail@kacst.edu.sa
2 Material Science Research Institute, King Abdulaziz City for Science and Technology, P.O. Box: 6086, Riyadh, Saudi Arabia; bshammari@kacst.edu.sa (B.A.A.); mfalotaibi@kacst.edu.sa (M.F.A.)
3 The National Center for Energy Storage Technologies, King Abdulaziz City for Science and Technology, P.O. Box: 6086, Riyadh, Saudi Arabia; gmotari@kacst.edu.sa (G.N.A.); Alenazey@kacst.edu.sa (F.S.A.)
4 Chemistry Department, College of Science, Jouf University, P.O. Box: 2014, Sakaka, Saudi Arabia ; amenad@ju.edu.sa (A.M.A.); agalharbi@ju.edu.sa (A.G.A.)
5 Electrical Engineer Department, Prince Sattam bin Abdulaziz University, P.O. Box: 173 Alkharj; Saudi Arabia; T.almoneef@psau.edu.sa
* Correspondence: bmalotaibi@kacst.edu.sa; Phone: +966-114883444 (ext. 1879)

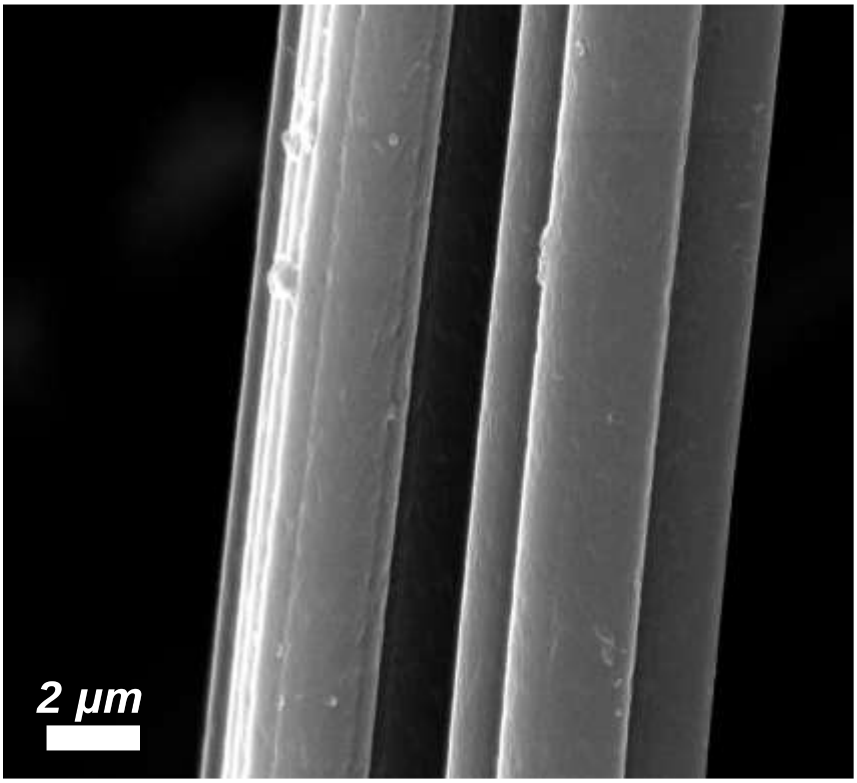

Figure S1. SEM image of OR-CF rough surface.

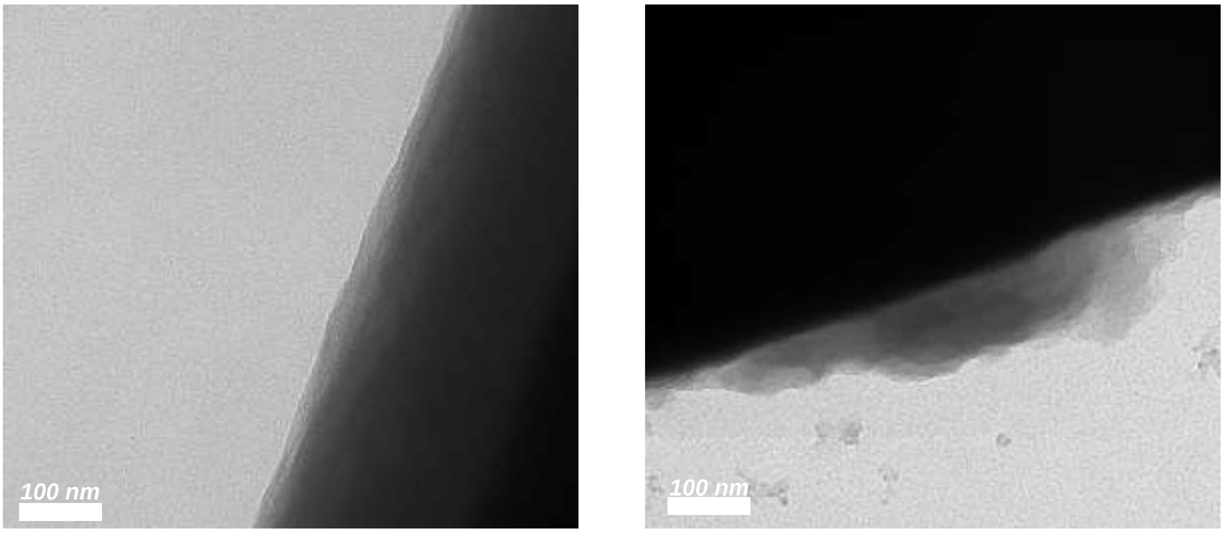

Figure S2. Low-magnification TEM at the edge of carbon felt fiber of untreated carbon felt CF (left) and treated carbon felt OR-CF (right).

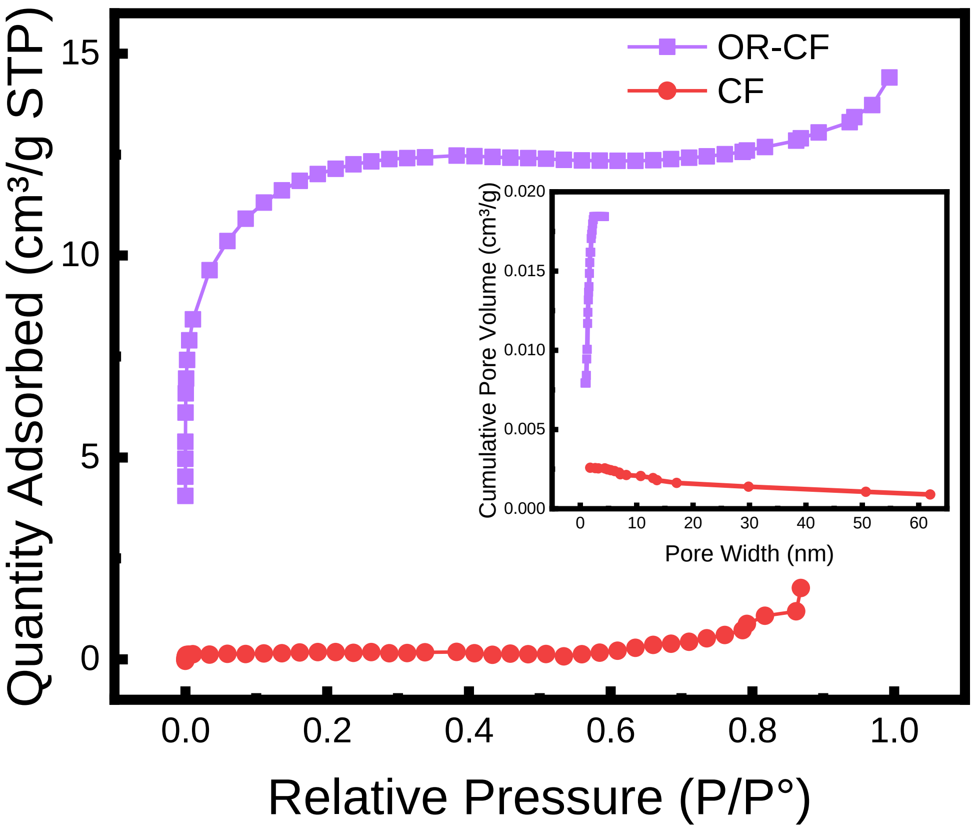

Figure S3. Nitrogen adsorption isotherm of CF and OR-CF at 77 K.

Table S1. FTIR corresponding functional groups contained corresponding to their peaks.

1057.01	C-O Stretching-	Primary alcohol or ether
1403.28	O-H bending-	Alcohol
1574.71	-C=C Stretching or N-H bending	cyclic alkene or Amine
1715.22	-COOH Stretching	Carboxylic acid

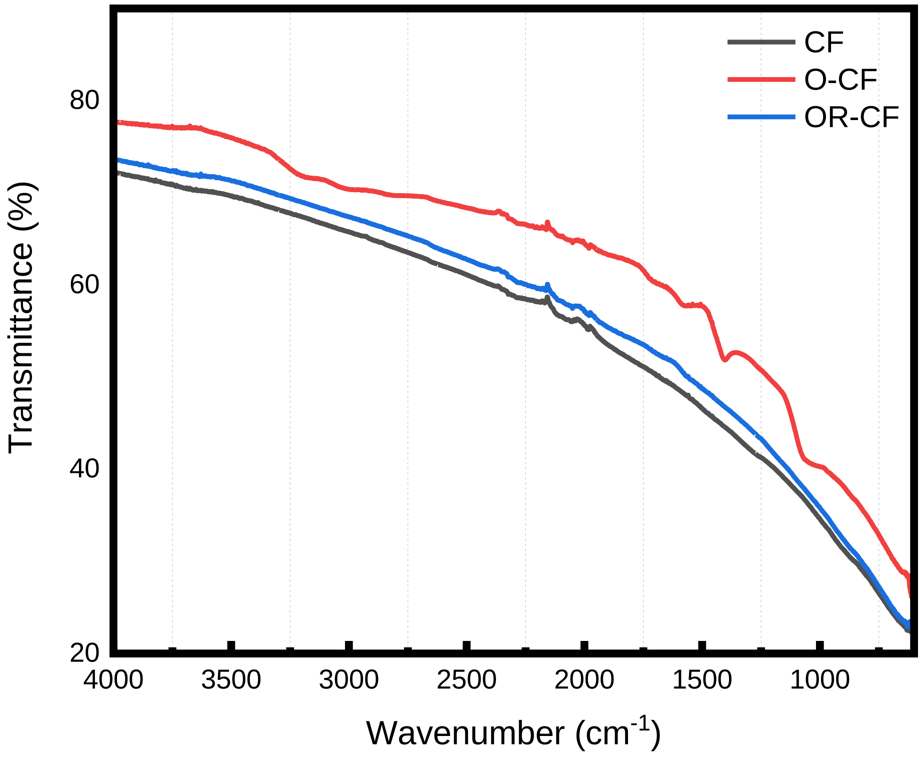

Figure S4. FTIR survey of CF, O-CF and OR-CF.

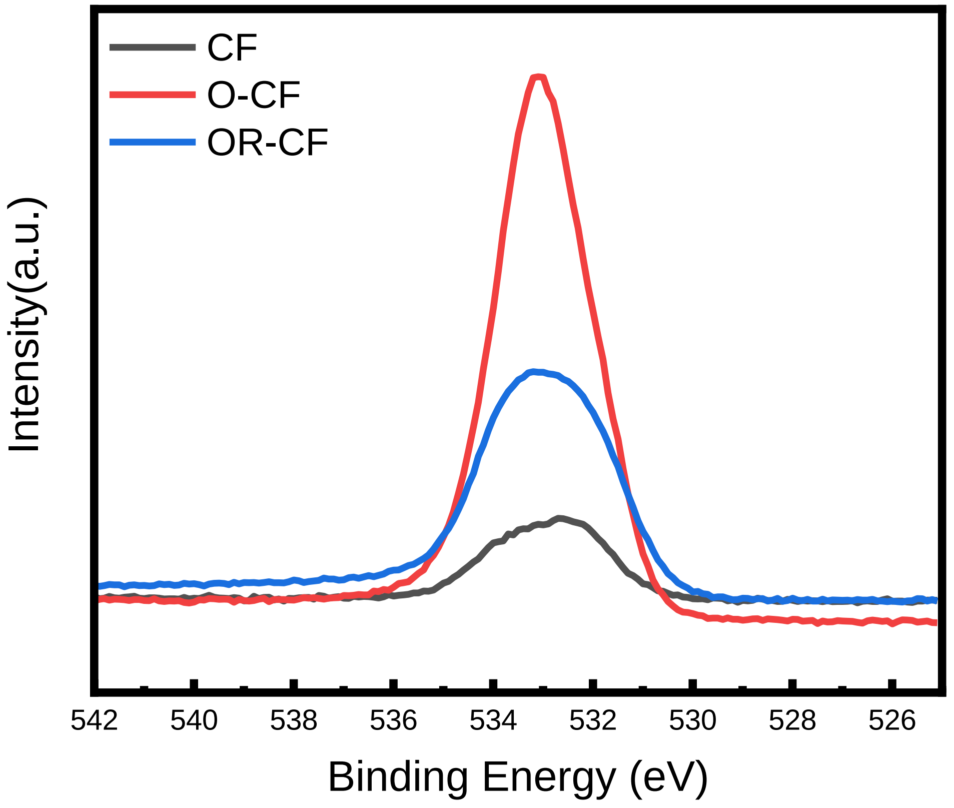

Figure S5. XPS O1s spectrum of CF, O-CF and OR-CF.

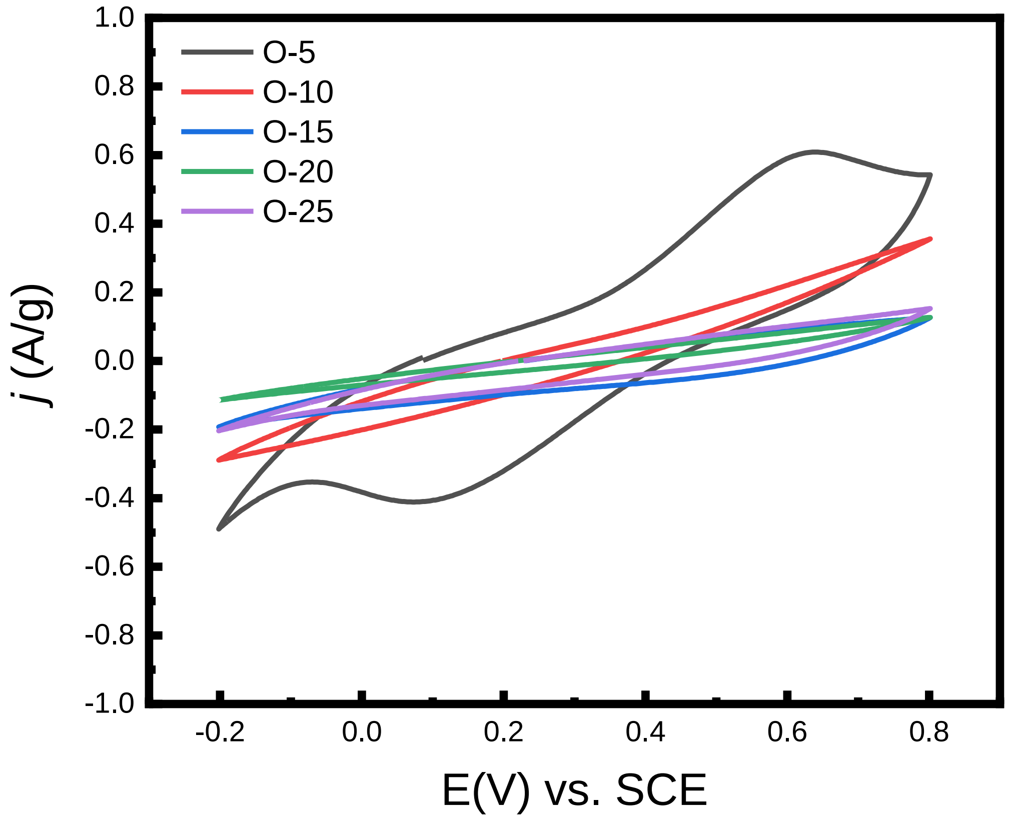

Figure S6. Cyclic voltammetry measurement of electrochemically oxidized carbon felt electrodes O-X (X: oxidizing time (5,10,15,20,25)).

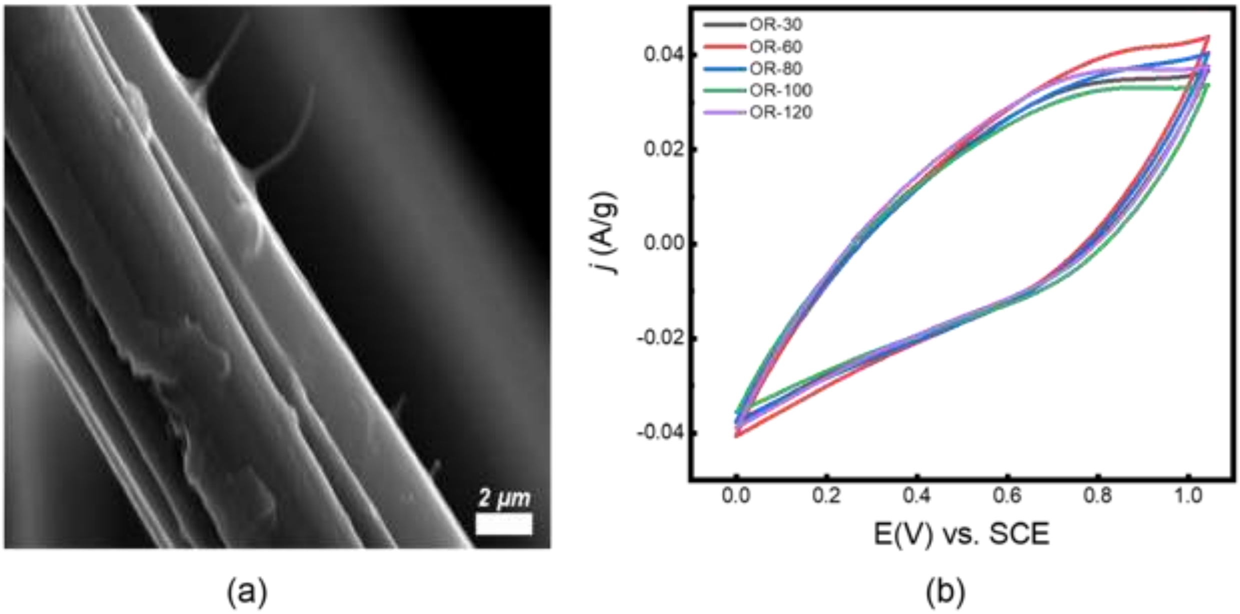

Figure S7. Extended electrochemical oxidizing conditions while constant reduction time of 30 minutes. (**a**) SEM image of OR-120 surface and (**b**) cyclic voltammetry of rest of the electrodes OR-X with X being corresponding treatment time in seconds.

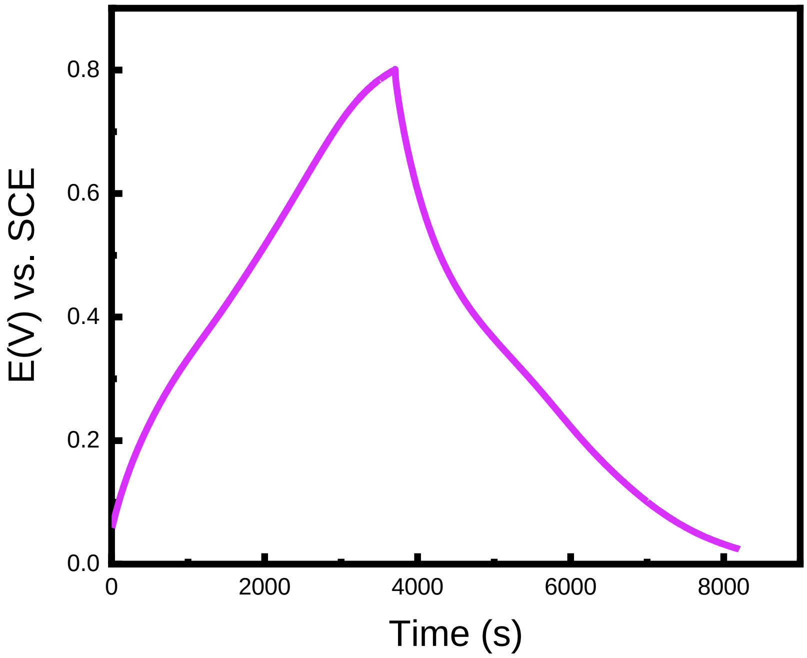

Figure S8. Galvanostatic charge/discharge measurements of OR-CF at 0.023 A g^{-1}.

Table S2. The corresponding circuit element values of impedance spectroscopy.

Element	Value
R1	7.921
CPE1-T	0.004515
CPE1-P	0.56218
R2	5.186
CPE2-T	0.23381
CPE2-P	0.8
